# Importance of double-resonance effects in two-photon absorption properties of Au_25_(SR)_18_^–^ †
†Electronic supplementary information (ESI) available. See DOI: 10.1039/c7sc00968b



**DOI:** 10.1039/c7sc00968b

**Published:** 2017-04-19

**Authors:** Zhongwei Hu, Lasse Jensen

**Affiliations:** a Department of Chemistry , The Pennsylvania State University , 104 Chemistry Building , University Park , Pennsylvania 16802 , USA . Email: jensen@chem.psu.edu

## Abstract

The two-photon absorption (TPA) cross-sections of small thiolate-protected gold clusters have been shown to be much larger than typical small organic molecules. In comparison with larger nanoparticles, their TPA cross-sections per gold atom are also found to be larger. Theoretical simulations have suggested that the large enhancement of these TPA cross-sections comes from a one-photon double-resonance mechanism. However, it remains difficult to simulate TPA cross-sections of thiolate-protected gold clusters due to their large system size and a high density of states. In this work, we report a time-dependent density functional theory (TDDFT) study of the TPA spectra of the Au_25_(SR)_18_^–^ cluster based on a damped response theory formalism. Damped response theory enables a consistent treatment of on- and off-resonance molecular properties even for molecules with a high density of states, and thus is well-suited for studying the TPA properties of gold clusters. Our results indicate that the one- and two-photon double-resonance effect is much smaller than previously found, and thus is unlikely to be the main cause of the large TPA cross-sections found experimentally. The effect of symmetry breaking of the Au_25_(SR)_18_^–^ cluster due to the ligands on the TPA cross-sections has been studied and was found to only slightly increase the cross-section. Furthermore, by comparing with larger nanoparticles we find that the TPA cross-section per gold atom scales linearly with the diameter of the particles, and that the Kerr non-linear response of the Au_25_(SR)_18_^–^ cluster is on the same order as that of bulk gold films.

## Abstract 2

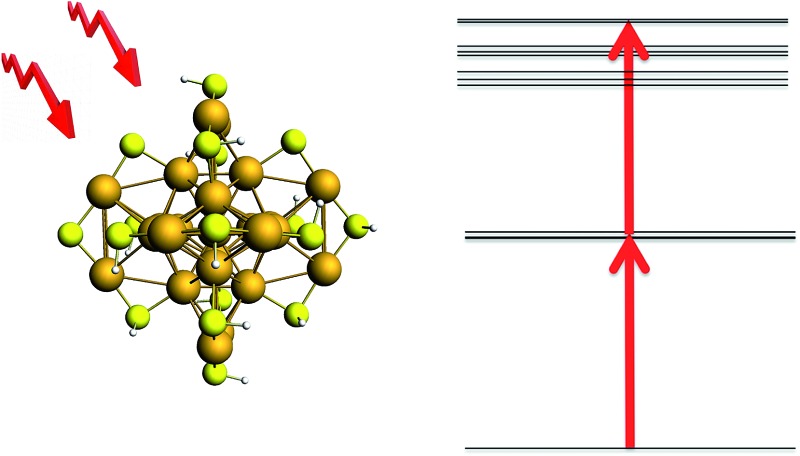
We show that double-resonance effects for Au_25_(SR)_18_^–^ are less pronounced and do not lead to significantly enhanced two-photon absorption cross-sections.

## 1 Introduction

Thiolate-protected gold nanoparticles have attracted significant interest in recent years due to their exceptional stability and applications in biomedicine, catalysis, electronics, photonics and sensing.^1–6^ While larger gold nanoparticles (>5 nm) are characterized by localized surface plasmon resonance, small gold clusters (<3 nm) exhibit molecular-like properties due to the quantum confinement effects.^3^ Although the exact boundary between molecular and plasmonic response has not been established, experiments have shown that a small nanoparticle containing only ∼330 gold atoms exhibits a plasmonic response.^7^ Simulations using time-dependent density functional theory (TDDFT) indicate that the boundary between molecular and plasmonic behaviors occurs for the Au_144_(SR)_60_ monolayer-protected cluster with a 1.5 nm core.^8^

Numerous gold clusters (Au_*n*_(SR)_*m*_) have been explored both experimentally and theoretically since the total structural determination of the nanoclusters Au_102_(SR)_44_ and Au_25_(SR)_18_^–^ using X-ray crystallography.^9–12^ Au_25_(SR)_18_^–^ is probably the most extensively studied cluster due to its extraordinary atomic packing structure and the well established structure–property relationship.^10–12^ The stability of the Au_25_(SR)_18_^–^ cluster can be understood by considering it to consist of a Au_13_^5+^ core surrounded by 6 anionic RS–(Au–SR)_2_^–^ units. According to the “super-atom” model,^13^ which is commonly used to understand the stability of gold clusters, this leads to a shell-closing of 8 electrons in the core that strengthens its stability. The existence of a crystal structure of the Au_25_(SR)_18_^–^ cluster has enabled a detailed correlation between its structure and optical properties through TDDFT simulations.^12,14,15^

There is a large amount of literature on the linear optical properties of small gold clusters. This is mainly because such properties are sensitive to the specific atomic arrangement, and thus can aid structural determination. Furthermore, understanding the linear properties of these small clusters provides insights into the emergence of the plasmonic response found in larger nanoparticles. The non-linear optical (NLO) properties of small gold clusters have also attracted attention due to their potential use in multiphoton imaging and optical limiting applications. Although much less work has been devoted to studying the NLO properties of thiolate-protected gold clusters in contrast to their linear counterparts, experimental two-photon absorption (TPA), non-linear transmission, hyper-Rayleigh scattering, and second- and third-harmonic generation measurements have been performed for the prototypical thiolate-protected Au_25_ cluster.^16–21^ However, few theoretical studies of the NLO properties of these small ligand-protected gold clusters are available in the literature due to a high computational burden.^22–24^

The most intriguing NLO property of the Au_25_(SR)_18_^–^ cluster is probably its large TPA cross-section in the communication wavelength region, which has been reported by Ramakrishna *et al.* ^16^ as 2700 GM for excitation at 1290 nm. This value is much larger than that of many organic chromophores, and promotes the Au_25_(SR)_18_^–^ cluster as a potentially well-qualified candidate for various NLO applications including biological imaging, nanolithography, and optical limiting.^17^ More interestingly, a very large TPA cross-section of 427 000 GM was also found for this cluster for excitation at 800 nm.^16^ Furthermore, the TPA cross-sections per gold atom for it and a few other small gold clusters were shown to exhibit a different size-scaling as compared to larger nanoparticles.^16^ To understand this unusual behavior, Day *et al.* ^24^ used TDDFT simulations based on the single residue of quadratic response functions to obtain the TPA cross-section of the Au_25_(SR)_18_^–^ cluster. These calculations suggested that a one-photon double-resonance effect could lead to the large TPA cross-sections observed experimentally. Recent work has also found that the optical Kerr response in 3 nm gold films is many orders of magnitude larger than that of the bulk metal.^25^ These results suggest that quantum size effects could lead to a significant third-order non-linear response for small gold clusters. However, simulations of resonance non-linear properties of metal clusters pose a significant challenge due to the high number of states contributing to the spectra, which raises the possibility of resonance effects that need to be dealt with carefully to avoid unphysically large response properties. This is particularly a problem when using standard response theory to calculate the molecular properties since the response functions diverge when optical frequencies, or the sum of them, equal an excitation energy.

In this work, we report simulated TPA spectra for the Au_25_(SR)_18_^–^ cluster using TDDFT. To avoid unphysical resonance effects, we will use a recently implemented damped cubic response formalism^26^ to calculate the TPA cross-sections. Damped response theory^26–30^ takes the broadening of electronic states into account and thus avoids the unphysical behaviors for molecular properties on resonance. In addition to TPA cross-sections, we will also characterize the resonance optical Kerr effect for the Au_25_(SR)_18_^–^ cluster. Both of these optical processes can be described by a third-order non-linear response tensor obtained using damped response theory. Our results show that the one-photon double resonance effect is smaller than previously found. We also find that the quantum size effects for the Au_25_(SR)_18_^–^ cluster do not lead to a significantly enhanced third-order non-linear response.

## 2 Theory

Considering the simultaneous absorbance process for two linearly polarized photons with identical energies,^31^ one can utilize the imaginary part of orientationally averaged third-order response properties to express the TPA cross-section (*σ* ^TPA^) as:^32^
1
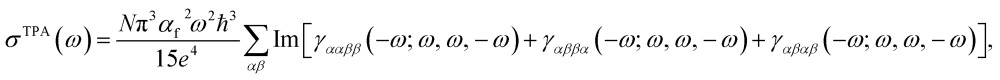
where *α*_f_ is the fine structure constant, *ω* is the incident frequency, and *N* is an integer value related to the experimental setup.^33^ In this work, *N* = 4 is used for all simulated TPA spectra and the unit of *σ* ^TPA^ is given as Göppert-Mayer (1 GM = 10^–50^ cm^4^ s per photon).^34^ The term *γ*(–*ω*; *ω*, *ω*, –*ω*) is denoted as *γ* ^IDRI^ in this work as its real part corresponds to the intensity-dependent refractive index (IDRI). The imaginary part of *γ* ^IDRI^ includes both saturated linear absorption and two-photon absorption, and in the traditional sum-over-states (SOS) approach the two processes can be related to the negative and two-photon terms (N-terms and T-terms), respectively.^35^ The N-terms provide large negative contributions to the *γ* tensors^36,37^ and correspond to purely one-photon processes, hence should not be considered when describing TPA. By eliminating the N-terms, one can obtain a reduced form for the IDRI, of which the imaginary part corresponds to the pure TPA process. This reduced IDRI, termed *γ* ^TPA^ in this work, can be given using the SOS expression as:2
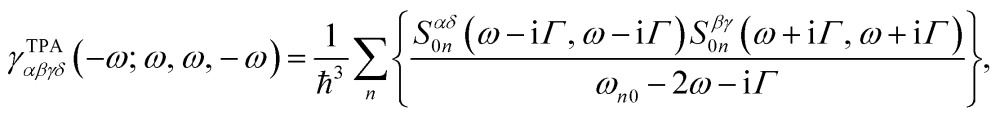
where *ω*_*n*0_ is the excitation energy of state *n*, *Γ* is the energy broadening parameter, and *S* ^*αβ*^ is the TPA transition moment that involves the transition dipole moments between the ground and excited states (〈0|*μ* ^*α*^|*m*〉) as well as two excited states (
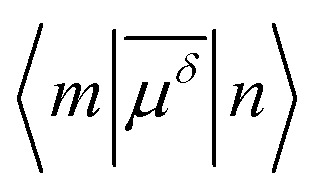
), *i.e.*
3
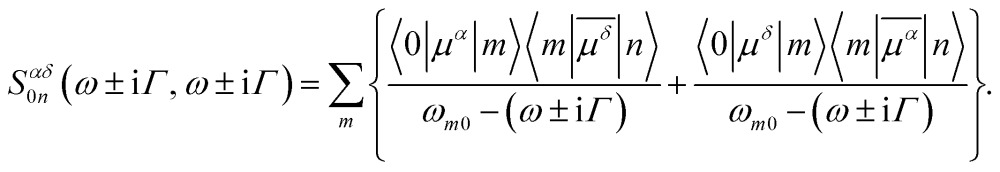



The corresponding expression for *γ* ^TPA^ in a damped response formalism has been previously reported by Kristensen *et al.* ^38^ and Hu *et al.* ^26^ The use of *γ* ^TPA^ avoids any negative TPA intensities caused by the pure one-photon processes and allows for appropriate *σ* ^TPA^ calculations in the presence of one- and two-photon double-resonance effects. Therefore, we adopt it for all TPA simulations in this work.

## 3 Computational details

All calculations in this work were carried out through a locally modified version of the Amsterdam Density Functional (ADF) 2014 program package.^39–41^ The starting geometry for the Au_25_(SH)_18_^–^ cluster is based on the crystal structure,^10,12^ and the initial atomic coordinates for the Au_25_(SH)_16_(SPh)_2_^–^ cluster were obtained from ref. 22. Geometry optimization was performed using the Becke–Perdew (BP86)^42,43^ XC functional with a small frozen-core triple-*ζ* polarized Slater-type (TZP) basis set from the ADF library. The BP86 XC functional with a large frozen-core TZP basis set was adopted to calculate the first- and third-order response properties. Scalar relativistic effects have been accounted for by means of the zeroth-order regular approximation (ZORA).^44,45^ Solvent effects are not included in the simulations, but good agreement between theory and experiment has been previously^12^ demonstrated for the absorption spectrum of the Au_25_(SR)_18_^–^ cluster. The finite lifetime of the electronic excited states is included phenomenologically using a damping parameter of 0.0034 a.u. (∼0.1 eV), which was previously found to be acceptable^29,30^ and also roughly the same as the Lorentzian fitting width used in ref. 12. The conversion factor to SI and cgs units for *γ* is:^46^ 1 a.u. = 7.0423 × 10^–54^ m^5^ V^–2^ = 5.0367 × 10^–40^ esu.

## 4 Results and discussion

**Fig. 1:**
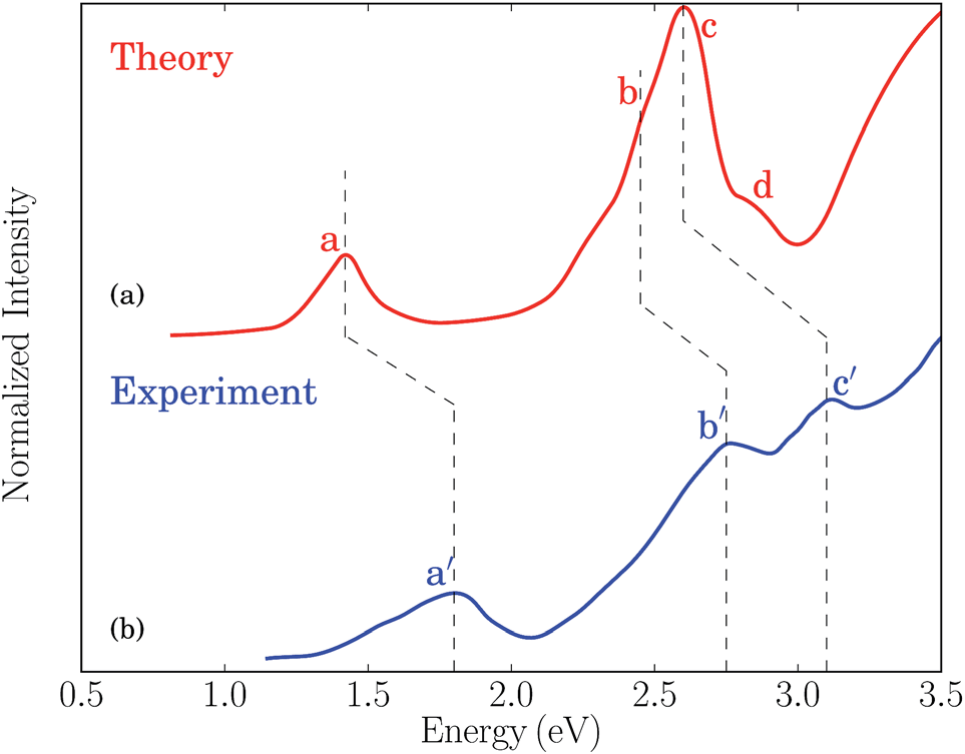
(a) Simulated absorption spectrum for the Au_25_(SR)_18_^–^ (R = H) cluster in the gas phase. (b) Experimental absorption spectrum for the Au_25_(SR)_18_^–^ (R = CH_2_CH_2_Ph) cluster in toluene taken from ref. 12.

The one-photon absorption spectrum of the Au_25_(SH)_18_^–^ cluster is characterized by three main bands found at 1.8, 2.8, and 3.1 eV, respectively.^12^ The lowest band is a HOMO to LUMO transition which can be characterized as an intraband transition (sp ← sp), the second band arises from mixed intraband (sp ← sp) and interband (sp ← d) transitions, while the third band arises predominantly from interband transitions (sp ← d).^12,14,15,47^ While initially the lowest transition at 1.8 eV was described in terms of the electronic and geometric structure of the Au_13_ core, recent work has shown that the optical absorption spectra are not separable into core and ligand contributions.^14^ A comparison between the simulated and experimental absorption spectra for the Au_25_(SR)_18_^–^ cluster is shown in Fig. 1. The simulated spectrum is obtained in the gas phase using the –SH group as the ligand while the experimental one is measured in toluene for the gold cluster passivated by the SCH_2_CH_2_Ph ligands.^12^

The theoretical spectrum consists of a major band at 1.43 eV and a broader band ranging from 2.2 → 3.1 eV. The former one (labeled as “a”) corresponds to the lowest band in the experimental spectrum at 1.8 eV (labeled as “a′”) and is primarily characterized by a HOMO to LUMO transition. The latter one has contributions from three groups of transitions at 2.4, 2.6, and 2.8 eV, labeled as “b”, “c” and “d”, respectively. The “b” band corresponds to the mixed intraband and interband experimental transitions at 2.8 eV (labeled as “b′”), and the “c” band corresponds to the experimental transitions at 3.1 eV (labeled as “c′”). Although the “d” band is not resolved in the experimental spectrum due to thermal broadening and its weak oscillator strength, low temperature measurements of the absorption spectrum have shown several additional bands above 3 eV.^48^ The red-shift of the simulated spectrum is likely a result of the neglection of solvent effects, the choice of XC functionals, and the simplified ligand used in the simulations.^49^ The band assignment presented here follows that of ref. 12, where a larger splitting of the “b” and “c” bands was obtained by using the SAOP XC potential. We refer to ref. 15 for a comprehensive discussion of the optical absorption of the thiolate-protected Au_25_ cluster.

**Fig. 2:**
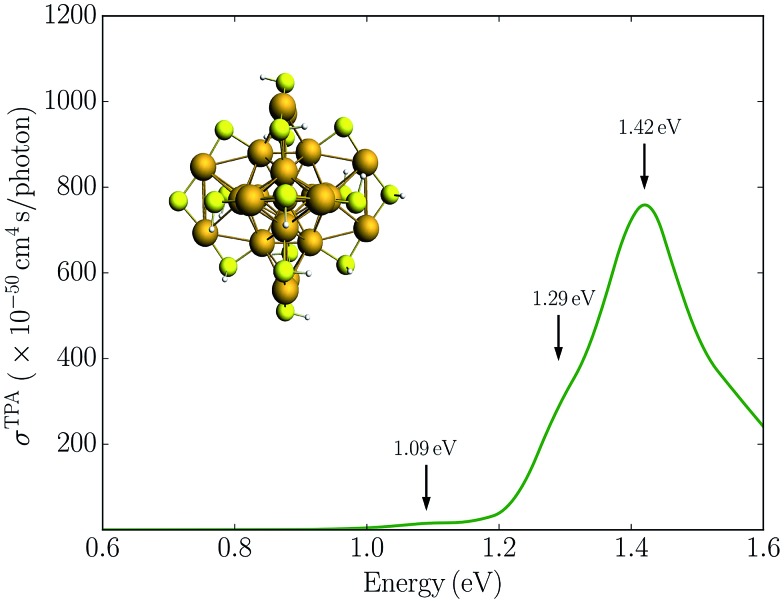
Simulated TPA spectrum for the Au_25_(SH)_18_^–^ clusters in the gas phase.

**Fig. 3:**
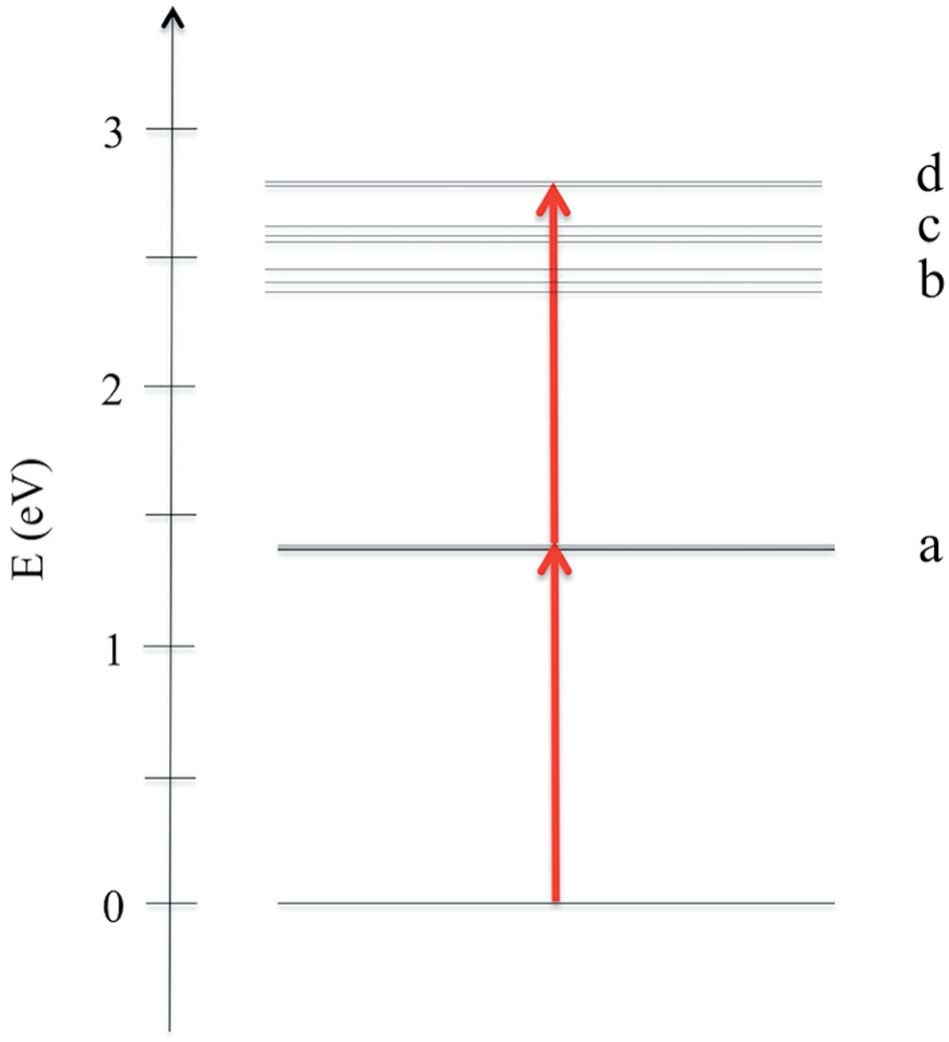
Schematic energy diagram showing the most important transitions for the Au_25_(SH)_18_^–^ cluster in the gas phase.

In Fig. 2 we plot the simulated TPA spectrum for the Au_25_(SH)_18_^–^ cluster as a function of the one-photon energy. The spectrum is dominated by a broad band at 1.4 eV with a shoulder at 1.3 eV and a weaker band at 1.1 eV. The weak low-energy band corresponds to two-photon excitations into a set of weaker states that are found as a shoulder to the “b” band in the one-photon absorption spectrum. The stronger shoulder at 1.3 eV in the two-photon spectrum corresponds to excitation into the strong “c” band in the one-photon absorption spectrum. Finally, the largest two-photon absorption cross-section is found around 753 GM for excitation into the “d” band at 1.4 eV. This *σ* ^TPA^ value is comparable to that of large organic TPA chromophores,^50^ mainly due to a double-resonance effect where the two-photon transition into the “d” band is enhanced by a one-photon resonance with the “a” band. We demonstrate this double-resonance effect by plotting a schematic energy diagram with the most important transitions in Fig. 3. From eqn (3), we see that the two-photon transition moments *S*_0*n*_ become large for photon energies near the one-photon transitions.

Experimentally, a huge TPA cross-section of 427 000 GM was found for a photon energy of 1.55 eV.^16^ Since this corresponds to excitation into the “a” band in the one-photon absorption spectrum, the huge TPA cross-section likely results from enhancement by one-photon processes, either a double-resonance effect or other effects such as excited state absorption. Previous simulations have found very large TPA cross-sections for small monolayer protected gold and silver clusters due to double-resonance effects.^24,51,52^ A TPA cross-section of 620 000 GM at a photon energy of 1.58 eV was reported for the Au_25_(SH)_18_^–^ cluster using B3LYP and a SDD-DZ basis set, with similar values found for the other functionals tested.^24^ The large TPA cross-section is in good agreement with the experimental observation and was attributed to resonance enhancement from the lowest excited state. However, in contrast to these huge TPA cross-sections found in the previous simulations, our results are several orders of magnitude smaller with the largest value found around 753 GM. Previous simulations used traditional quadratic response theory within a TDDFT formalism to simulate the TPA cross-section, which can produce unphysical large TPA cross-sections due to double-resonance effects.^26,38,53^ A major advantage of using damped response theory is that the two-photon transition moments remain finite even if in the vicinity of one-photon resonances, and thus can correctly describe this double-resonance effect.^24^

Experimentally, it was also found that the TPA cross-sections per gold atom for the small gold clusters were significantly larger than those for larger nanoparticles, following a different size dependence.^16^ This was attributed to quantum size effects and indicated a transition from small clusters characterized in terms of discrete transitions to larger nanoparticles characterized in terms of plasmon resonances. For the Au_25_(SH)_18_^–^ cluster, a TPA cross-section per gold atom of 17 080 GM was reported^16^ while we find a much smaller value of 30 GM per gold atom. However, our results compare well with the expected value based on the size-scaling observed for the TPA cross-sections of the larger nanoparticles. This is illustrated in the ESI,† where we plot the experimental *σ* ^TPA^/gold as a function of the size of the Au_976_ (3.0 nm) and the Au_2406_ (4.0 nm) nanoparticles^16^ compared with the simulated results for the Au_25_(SH)_18_^–^ cluster.

The experiments also reported a large TPA cross-section of 2700 GM for a photon energy of 0.96 eV, corresponding to a two-photon excitation into the “a′” band.^16^ As this is the lowest one-photon band, the possible double-resonance effects can be ruled out. Considering the fact that the orientation of the ligands with respect to the Au_25_S_18_ core makes the entire cluster roughly centrosymmetric,^10–12^ one should expect the lowest excited state to be one-photon allowed but two-photon forbidden. This is consistent with our simulations, where no significant TPA cross-section is found for excitation into the “a” band. This is also in agreement with the results reported by Day *et al.* ^24^ The large TPA cross-section at 0.96 eV was speculated by Day *et al.* ^24^ as the result of the tail of the very large TPA cross-section for excitation into the “d” band at 1.4 eV. However, that is not seen in our simulations.

**Fig. 4:**
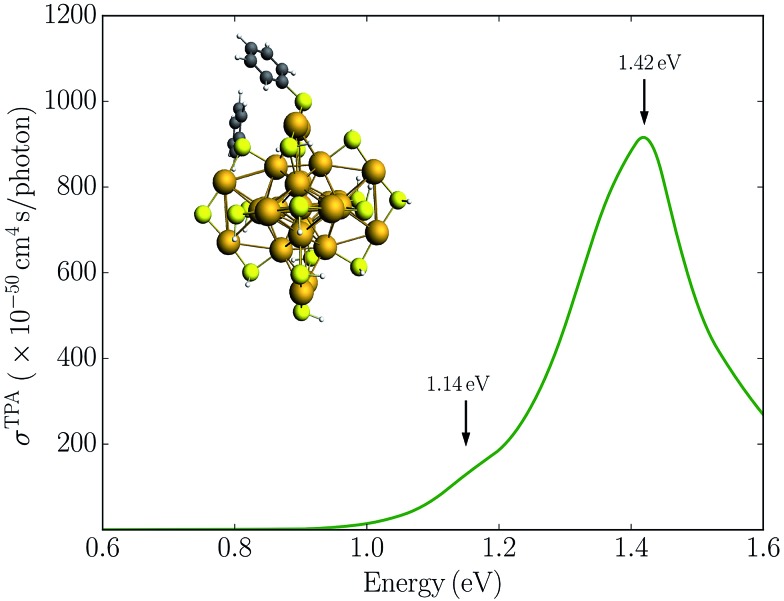
Simulated TPA spectrum for the Au_25_(SH)_16_(SPh)_2_^–^ cluster in the gas phase.

Alternatively, symmetry breaking of the ligand shell could lead to an increased TPA cross-section. Previous work has demonstrated that such symmetry breaking leads to the observation of hyper-Rayleigh scattering of the Au_25_(SR)_18_^–^ cluster which otherwise would be symmetry forbidden due the centrosymmetry.^19,22^ To investigate the symmetry breaking effects on the TPA cross-section, we also considered the Au_25_(SH)_16_(SPh)_2_^–^ cluster where two phenyl groups have been substituted. This structure is taken from previous work investigating the symmetry breaking effects on the first hyperpolarizabilities.^22^ The TPA spectrum for the Au_25_(SH)_16_(SPh)_2_^–^ cluster is shown in Fig. 4. In comparison to the Au_25_(SH)_18_^–^ cluster, the addition of the two phenyl groups leads to slightly larger TPA cross-sections with a maximum of 905 GM. However, no significant TPA cross-section is found for excitation into the “a” band, and thus symmetry breaking is unlikely to be the reason for the large TPA cross-section observed experimentally.

**Fig. 5:**
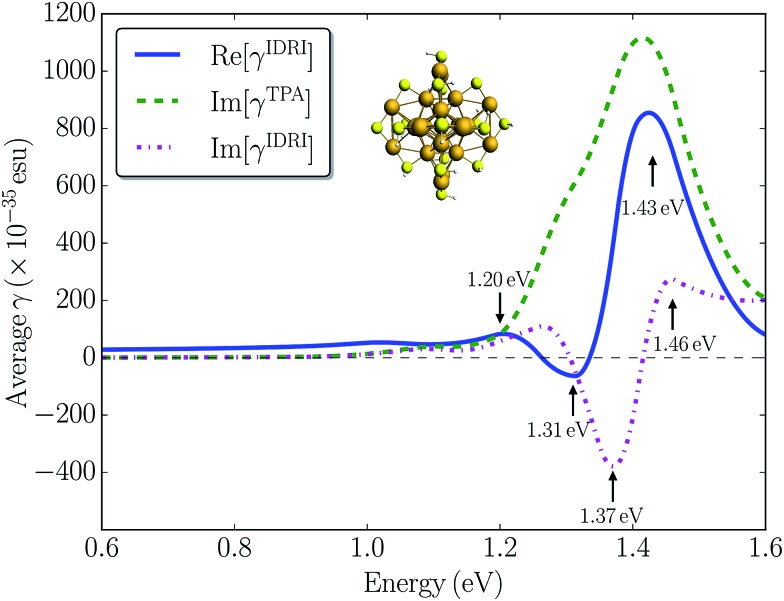
Calculated Re[*γ* ^IDRI^] (blue solid line), Im[*γ* ^TPA^] (green dashed line), and Im[*γ* ^IDRI^] (magenta dash-dotted line) for the Au_25_(SH)_18_^–^ cluster in the gas phase. The black dashed line shows a value of 0 esu.

To investigate the importance of other one-photon resonance enhancements in the third-order non-linearity of Au_25_(SH)_18_^–^, we simulated the optical Kerr response corresponding to the *γ*(–*ω*; *ω*, *ω*, –*ω*) tensor with all one-photon terms included. The real part of the optical Kerr response is related to the IDRI and its imaginary part corresponds to two-photon and saturated one-photon absorption processes. In Fig. 5 we plot both Re[*γ* ^IDRI^] and Im[*γ* ^IDRI^] for the Au_25_(SH)_18_^–^ cluster as a function of the one-photon energy, together with the contribution arising only from two-photon absorption, *i.e.* Im[*γ* ^TPA^]. The Re[*γ* ^IDRI^] curve is characterized by a large positive band at 1.43 eV and a small negative band at 1.31 eV. In contrast, a large negative band at 1.37 eV with a small positive band at 1.46 eV mainly constitutes the Im[*γ* ^IDRI^] curve. Im[*γ* ^TPA^] is much larger than Im[*γ* ^IDRI^] demonstrating a strong destructive interference between the one- and two-photon processes. This seems to indicate that the one-photon processes are more dominant compared to the two-photon processes, and could be the reason that only relatively modest TPA cross-sections are found for the Au_25_(SH)_18_^–^ cluster. The magnitude of the optical Kerr response determined here is comparable to that of the pure electronic response in organic molecules.^54^ Although there are no direct measurements of the optical Kerr response of small gold clusters, Qian *et al.* ^25^ reported the optical Kerr response of 3 nm gold films. They found a large Kerr susceptibility of 2.06 × 10^–15^ m^2^ V^–2^, which is about four orders of magnitude larger than that determined for bulk gold films. The large non-linear response observed was attributed to quantum size effects in the thin gold film. Here we calculated |*χ* ^3^| to be 3.26 × 10^–19^ m^2^ V^–2^ for the Au_25_(SH)_18_^–^ cluster by assuming a diameter of ∼1.1 nm. Our calculated value is comparable to the bulk value of 9.11 × 10^–19^ m^2^ V^–2^ for a 15 nm thick gold film reported in the same work, but much smaller than the thin film result.^25^ Thus, in general we do not find that quantum size effects lead to a significantly enhanced non-linear response in the small monolayer protected clusters.

Although our simulations show that the TPA cross-sections for the Au_25_(SH)_18_^–^ cluster are enhanced by a double-resonance effect, they are much smaller than what has been indicated by previous simulations or found experimentally. In general, our results seem to suggest that quantum size effects in these small Au_25_(SR)_18_^–^ clusters do not lead to extremely large TPA cross-sections. However, it is important to note that the results presented here are sensitive to the exact value used for the energy broadening parameter (*Γ*). In this work, *Γ* was chosen to match the experimental absorption spectrum. Using a smaller *Γ* value would lead to larger TPA cross-sections, but one would need unphysical small values to match the experimental TPA cross-sections. Finally, the TDDFT simulations presented here all employ an adiabatic approximation for the exchange–correlation kernels. Previously, it was shown that such a typical approximation could cause spurious pole effects near one-photon resonances.^26^ For small molecules, these spurious poles lead to significantly larger TPA cross-sections, yet the behavior is still unknown for systems like the Au_25_(SR)_18_^–^ clusters that have a high density of states.

## 5 Conclusions

In summary, we have reported first-principles simulations of TPA spectra for two thiolate-protected Au_25_ clusters based on a damped cubic response formalism within TDDFT. We find that the calculated TPA cross-sections are much smaller than their experimental counterparts, which indicates that the previously suggested one- and two-photon double resonance effect is unlikely to be the only cause for the large TPA intensities reported experimentally. The calculation of TPA cross-sections on a per gold atom basis, as well as the Kerr non-linear responses, is in-line with those expected from larger nanoparticles. Symmetry breaking was shown to only lead to small enhancements of the TPA cross-sections. Overall, this work represents the first cubic response theory approach to TPA simulations of the Au_25_(SR)_18_^–^ clusters, and shows that quantum size effects do not lead to a significantly enhanced third-order non-linear response. This is in agreement with the molecular origin of the electronic transitions in the small gold clusters.

## References

[cit1] Daniel M.-C., Astruc D. (2004). Chem. Rev..

[cit2] Häkkinen H. (2008). Chem. Soc. Rev..

[cit3] Jin R. (2010). Nanoscale.

[cit4] Dreaden E. C., Alkilany A. M., Huang X., Murphy C. J., El-Sayed M. A. (2012). Chem. Soc. Rev..

[cit5] Fernando A., Weerawardene K. L. D. M., Karimova N. V., Aikens C. M. (2015). Chem. Rev..

[cit6] Jin R., Zeng C., Zhou M., Chen Y. (2016). Chem. Rev..

[cit7] Qian H., Zhu Y., Jin R. (2012). Proc. Natl. Acad. Sci. U. S. A..

[cit8] Malola S., Lehtovaara L., Enkovaara J., Häkkinen H. (2013). ACS Nano.

[cit9] Jadzinsky P. D., Calero G., Ackerson C. J., Bushnell D. A., Kornberg R. D. (2007). Science.

[cit10] Heaven M. W., Dass A., White P. S., Holt K. M., Murray R. W. (2008). J. Am. Chem. Soc..

[cit11] Akola J., Walter M., Whetten R. L., Häkkinen H., Grönbeck H. (2008). J. Am. Chem. Soc..

[cit12] Zhu M., Aikens C. M., Hollander F. J., Schatz G. C., Jin R. (2008). J. Am. Chem. Soc..

[cit13] Walter M., Akola J., Lopez-Acevedo O., Jadzinsky P. D., Calero G., Ackerson C. J., Whetten R. L., Grönbeck H., Häkkinen H. (2008). Proc. Natl. Acad. Sci. U. S. A..

[cit14] Aikens C. M. (2008). J. Phys. Chem. C.

[cit15] Aikens C. M. (2009). J. Phys. Chem. A.

[cit16] Ramakrishna G., Varnavskit O., Kim J., Lee D., Goodson T. (2008). J. Am. Chem. Soc..

[cit17] Yau S. H., Varnavski O., Theodore Goodson I. (2013). Acc. Chem. Res..

[cit18] Philip R., Chantharasupawong P., Qian H., Jin R., Thomas J. (2012). Nano Lett..

[cit19] Russier-Antoine I., Bertorelle F., Vojkovic M., Rayane D., Salmon E., Jonin C., Dugourd P., Antoine R., Brevet P.-F. (2014). Nanoscale.

[cit20] Knoppe S., Vanbel M., van Cleuvenbergen S., Vanpraet L., Bürgi T., Verbiest T. (2015). J. Phys. Chem. C.

[cit21] Olesiak-Banska J., Waszkielewicz M., Matczyszyn K., Samoc M. (2016). RSC Adv..

[cit22] Knoppe S., Häkkinen H., Verbiest T. (2015). J. Phys. Chem. C.

[cit23] Van Steerteghem N., Van Cleuvenbergen S., Deckers S., Kumara C., Dass A., Häkkinen H., Clays K., Verbiest T., Knoppe S. (2016). Nanoscale.

[cit24] Day P. N., Nguyen K. A., Pachter R. (2010). J. Chem. Theory Comput..

[cit25] Qian H., Xiao Y., Liu Z. (2016). Nat. Commun..

[cit26] Hu Z., Autschbach J., Jensen L. (2016). J. Chem. Theory Comput..

[cit27] Norman P., Bishop D. M., Jensen H. J. A., Oddershede J. (2001). J. Chem. Phys..

[cit28] Norman P., Bishop D. M., Jensen H. J. A., Oddershede J. (2005). J. Chem. Phys..

[cit29] Jensen L., Autschbach J., Schatz G. C. (2005). J. Chem. Phys..

[cit30] Hu Z., Autschbach J., Jensen L. (2014). J. Chem. Phys..

[cit31] McClain W. M. (1971). J. Chem. Phys..

[cit32] Silverstein D. W., Jensen L. (2012). J. Chem. Phys..

[cit33] Beerepoot M. T. P., Friese D. H., List N. H., Kongsted J., Ruud K. (2015). Phys. Chem. Chem. Phys..

[cit34] Göppert-Mayer M. (1931). Ann. Phys..

[cit35] Ensley T. R., Hu H., Reichert M., Ferdinandus M. R., Peceli D., Hales J. M., Perry J. W., Li Z., Jang S.-H., Jen A. K.-Y., Marder S. R., Hagan D. J., Stryland E. W. V. (2016). J. Opt. Soc. Am. B.

[cit36] Pérez-Moreno J., Kuzyk M. G. (2005). J. Chem. Phys..

[cit37] Pérez-Moreno J., Clays K., Kuzyk M. G. (2008). J. Chem. Phys..

[cit38] Kristensen K., Kauczor J., Thorvaldsen A. J., Jørgensen P., Kjærgaard T., Rizzo A. (2011). J. Chem. Phys..

[cit39] te Velde G., Bickelhaupt F. M., Baerends E. J., Fonseca Guerra C., van Gisbergen S. J. A., Snijders J. G., Ziegler T. (2001). J. Comput. Chem..

[cit40] Fonseca Guerra C., Snijders J. G., te Velde G., Baerends E. J. (1998). Theor. Chem. Acc..

[cit41] Baerends E., Ziegler T., Autschbach J., Bashford D., Bérces A., Bickelhaupt F., Bo C., Boerrigter P., Cavallo L., Chong D., Deng L., Dickson R., Ellis D., van Faassen M., Fan L., Fischer T., Guerra C. F., Franchini M., Ghysels A., Giammona A., van Gisbergen S., Götz A., Groeneveld J., Gritsenko O., Grüning M., Gusarov S., Harris F., van den Hoek P., Jacob C., Jacobsen H., Jensen L., Kaminski J., van Kessel G., Kootstra F., Kovalenko A., Krykunov M., van Lenthe E., McCormack D., Michalak A., Mitoraj M., Morton S., Neugebauer J., Nicu V., Noodleman L., Osinga V., Patchkovskii S., Pavanello M., Philipsen P., Post D., Pye C., Ravenek W., Rodríguez J., Ros P., Schipper P., van Schoot H., Schreckenbach G., Seldenthuis J., Seth M., Snijders J., Solà M., Swart M., Swerhone D., te Velde G., Vernooijs P., Versluis L., Visscher L., Visser O., Wang F., Wesolowski T., van Wezenbeek E., Wiesenekker G., Wolff S., Woo T. and Yakovlev A., Amsterdam Density Functional, 2014.

[cit42] Becke A. D. (1988). Phys. Rev. A.

[cit43] Perdew J. P. (1986). Phys. Rev. B: Condens. Matter Mater. Phys..

[cit44] van Lenthe E., Baerends E. J., Snijders J. G. (1993). J. Chem. Phys..

[cit45] van Lenthe E., Baerends E. J., Snijders J. G. (1994). J. Chem. Phys..

[cit46] Shelton D. P., Rice J. E. (1994). Chem. Rev..

[cit47] Aikens C. M. (2011). J. Phys. Chem. Lett..

[cit48] Devadas M. S., Bairu S., Qian H., Sinn E., Jin R., Ramakrishna G. (2011). J. Phys. Chem. Lett..

[cit49] Zhang H.-F., Stender M., Zhang R., Wang C., Li J., Wang L.-S. (2004). J. Phys. Chem. B.

[cit50] Terenziani F., Katan C., Badaeva E., Tretiak S., Blanchard-Desce M. (2008). Adv. Mater..

[cit51] Day P. N., Pachter R., Nguyen K. A., Bigioni T. P. (2016). J. Phys. Chem. A.

[cit52] Sanader Ž., Krstić M., Russier-Antoine I., Bertorelle F., Dugourd P., Brevet P.-F., Antoine R., Bonačić-Koutecký V. (2016). Phys. Chem. Chem. Phys..

[cit53] Parker S. M., Roy S., Furche F. (2016). J. Chem. Phys..

[cit54] Vigil S. R., Kuzyk M. G. (2001). J. Opt. Soc. Am. B.

