# Supplementary material for: Importance of double-resonance effects in two-photon absorption properties of Au25(SR)18 –
Source: Chem Sci. 2017 Apr 19;8(6):4595–601. doi: 10.1039/c7sc00968b (PMC5471455; doi:10.1039/c7sc00968b)
Supplement: Supplementary file 1 [file SC-006-C7SC00968B-s001.pdf]

Electronic Supplementary Material (ESI) for Chemical Science.
This journal is © The Royal Society of Chemistry 2017

Supporting Information

Importance of Double-Resonance Effects in Two-Photon Absorption Properties of $Au_{25}(SR)_{18}^{-}$

Zhongwei Hu and Lasse Jensen*

The Pennsylvania State University, Department of Chemistry, 104 Chemistry Building, University Park, PA 16802, USA

E-mail: jensen@chem.psu.edu

TPA cross sections on a per gold atom basis as a function of the cluster size

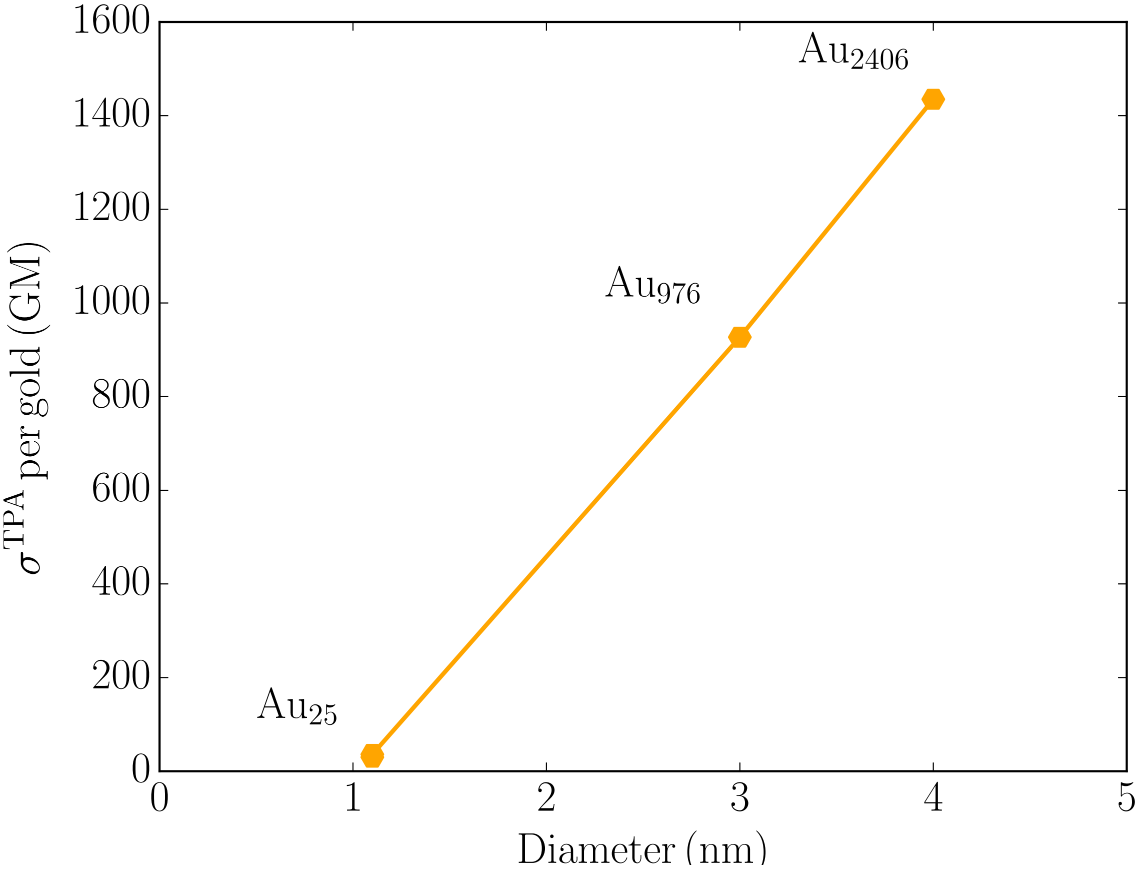

Figure 1: TPA cross section on a per atom basis for three different gold species. The values for the $Au_{25}(SH)_{18}^{-}$ cluster is based on simulations while those for the Au_{976} and the Au_{2406} nanoparticles were obtained using the experimental data from Ref. 1.

References

(1) Ramakrishna, G.; Varnavskit, O.; Kim, J.; Lee, D.; Goodson, T. Quantum-Sized Gold Clusters as Efficient Two-Photon Absorbers. *J. Am. Chem. Soc.* **2008**, *130*, 5032.
